# Manual of Ophthalmic Surgery and Medicine

**Published:** 1900-12

**Authors:** 


**Manual of Ophthalmic Surgery and Medicine.** By WALTER H. H. JESSOP, M.B. Pp. xiv., 469. London: J. & A. Churchill. 1898.

Among the many excellent handbooks devoted to ophthalmic practice, the one before us deserves to be well recommended. The book is dedicated to the students of St. Bartholomew's Hospital, and it is probably chiefly for the use of students that it was written. Its matter is well arranged, clear and concise, and it is accurate and up to date. The symptoms and progress of a disease, its causation and pathology, are adequately dealt with before any mention of its treatment is made. A short but sufficient description of the anatomy of each structure of the eyeball is given, before the diseases to which that portion is liable are described.

The chapters on the pupil, and on the affections of the ocular muscles, are amongst the best in the book.

Both the student of medicine and the medical practitioner will find Jessop's *Manual* well adapted towards an acquisition of a knowledge of the eye and its diseases, or for reference in difficulties as to the diagnosis or the treatment of such diseases.

